# Research Progress on Pharmacological Effects of Key Pharmacodynamic Material Basis Components of *Schisandrae chinensis* Fructus

**DOI:** 10.4014/jmb.2602.02009

**Published:** 2026-07-29

**Authors:** Hang Pan, Hongxuan Yu, Rui Liu, Liru Zhou, Zehua Wang, Qiong Liu, Guizhen Wang

**Affiliations:** 1 College of Biological and Food Engineering, Jilin Engineering Normal University, Changchun 130052, P. R. China; 2 Engineering Research Center of Microecological Vaccines (Drugs) for Major Animal Diseases, Ministry of Education, Jilin Agricultural University, Changchun 130118, P. R. China

**Keywords:** *Schisandra chinensis*, Bioactive components, Pharmacological effects, Mechanism of action

## Abstract

*Schisandra chinensis* Baill. and its dried ripe fruit, *Schisandrae chinensis* Fructus, are typical medicinal and edible resources in traditional Chinese medicine. Modern studies indicate that this medicinal material contains lignans, polysaccharides, volatile oils, triterpenoids, flavonoids, and other constituents, among which lignans are the most characteristic pharmacodynamic material basis. Based on the literature covered in this review, this paper summarizes the main bioactive components and structural features of *S. chinensis* and reviews the pharmacological evidence for antioxidation, anti-inflammatory activity, anti-tumor activity, sedative-hypnotic effects, hepatoprotection, neuroprotection, and metabolic regulation. The currently direct compound-specific evidence is relatively clearer for schisandrin B, schisandrol A/B, and schisanhenol in selected experimental models, whereas several other reported activities still rely mainly on extract-level studies, network pharmacology, or indirect inference. This review aims to provide an evidence-based reference for clarifying the pharmacodynamic material basis, improving quality evaluation, and guiding further mechanistic and clinical studies of *S. chinensis* Fructus.

## Introduction

*Schisandra chinensis* Baill. (Schisandraceae), commonly known as “Beiwuweizi”, is the dried ripe fruit of the plant and has long been used as both medicine and food. It was listed as a superior herb in the Shennong Ben Cao Jing, reflecting its long history of medicinal application [1, 2]. In traditional Chinese medicine, it is sour and sweet in flavor and warm in nature, and enters the lung, heart, and kidney meridians. It is traditionally used for chronic cough and asthma due to deficiency, spermatorrhea, enuresis and frequent urination, chronic diarrhea, spontaneous and night sweating, thirst caused by fluid depletion, as well as palpitations and insomnia [1-3]. In modern practice and ethnobotanical investigations, *S. chinensis* is also widely used in functional foods and traditional Chinese medicine, further highlighting its dual medicinal and edible value [1, 2, 4].

The broad pharmacological effects of *S. chinensis* are closely related to its complex chemical composition. Lignans are the most characteristic constituents and are generally regarded as the core pharmacodynamic material basis, while polysaccharides, volatile oils, triterpenoids, flavonoids, and phenolic acids also contribute to its biological activities [1, 2, 5-9]. In recent years, analytical chemistry, network pharmacology, metabolomics, and pharmacodynamic validation studies have considerably advanced understanding of its active components and mechanisms, with reported activities involving hepatoprotection [10-13], sedative-hypnotic or neurotransmitter-regulating effects [14-18], anti-tumor activity [19], anti-muscle atrophy [20, 21], glucose and lipid metabolism regulation [22, 23], tissue repair [24], ameliorating depressive disorders [25], and intervention in complex diseases such as Alzheimer's disease [26-30] and diabetic nephropathy [22, 31]. However, the level of evidence is not uniform across individual compounds, and some claimed mechanisms are still supported mainly by extract-level data or indirect prediction. Therefore, this review focuses on the main bioactive components and critically summarizes the available pharmacological evidence, with particular attention to direct compound-specific validation and current research gaps.

### Main Bioactive Components and Structural Features

Modern phytochemical research confirms that the biological activities of *S. chinensis* originate from a complex system of chemical constituents. Lignans are the core active components, complemented by polysaccharides, volatile oils, triterpenoids, flavonoids, and phenolic acids [1, 2, 5, 6, 32, 33]. The lignans are characterized by a dibenzocyclooctadiene (DBCOD) skeleton, an eight-membered ring structure that serves as the key pharmacophore responsible for their therapeutic effects. Consequently, these lignans are collectively referred to as dibenzocyclooctadiene lignans. Representative compounds include schisandrin, schisantherin A, schisandrol A, schisandrol B, schisandrin A, schisandrin B, schisanhenol, gomisin D, angeloylgomisin H, angeloylgomisin Q, gomisin G, gomisin K, benzoylgomisin H, and gomisin J [2, 12, 16]. The content and distribution of these constituents vary across medicinal materials and samples, which also underlies the need for component-based quality control [1, 2, 33]. All chemical structures of small molecular compounds in this study were retrieved from the PubChem database (https://pubchem.ncbi.nlm.nih.gov/). The main bioactive components and their chemical structures are shown in Table 1.

**Lignans.** Lignans are the most representative active components of *Schisandra chinensis* and belong to the dibenzocyclooctadiene lignan class. To date, dozens of lignan compounds have been isolated and identified from this plant. Analytical methods such as LC-MS/MS, HPLC-MS, magnetic separation coupled with mass spectrometry, and aqueous two-phase/ultrasound-assisted extraction have been applied to the identification, quality evaluation, and enrichment of Schisandra lignans [14, 34-36]. Common lignans include schisandrol A (**6**), schisantherin A (**8**), schisandrin A (**7**), schisandrin B (**5**), schisandrol B (**9**), schisandrin C (**10**), and schisanhenol (**12**) [2, 12, 16, 37]. Schisandrol A (**6**) has been specifically reviewed in terms of its preparation, biological activities, and pharmacokinetics [16]. Because lignan composition varies with species, origin, and medicinal materials, these compounds are also used as important markers in the authentication and quality control of Schisandra-related preparations [34, 36].

**Polysaccharides.** *S. chinensis* polysaccharides represent another important class of bioactive macromolecules. The extraction method directly influences the yield and purity of these polysaccharides, and combining multiple methods typically enhances the extraction rate. Studies have shown that SCPs are mainly composed of monosaccharides such as glucose, mannose, rhamnose, galactose, galacturonic acid, and arabinose [5, 6]. And polysaccharides account for approximately 10% of *S. chinensis* [38]. Different extraction methods will affect the molecular structure of SCPs and thereby alter its biological activity. Available evidence indicates that SCPs possess a wide range of biological activities, including immunomodulatory, antioxidant, hepatoprotective, anti-inflammatory, and hypoglycemic effects [5, 6, 23].

**Volatile oils.** The essential oil of *S. chinensis* consists of volatile components extracted from its fruit. Gas chromatography-mass spectrometry analyses have shown that these volatile fractions are rich in terpenoids and related constituents [18, 19]. Available studies indicate that SEO exhibits neuroprotective and anti-inflammatory activities in experimental models, and can ameliorate cognitive impairment in mouse models by reducing neuroinflammation through the NF-κB/MAPK pathway [9].

**Triterpenoids and other components.** The leaves of *S. chinensis*, share a high degree of compositional similarity with *S. chinensis* [39]. Structurally novel nortriterpenoids, such as Wunorlactones A-G, have been isolated from the stems and leaves of *S. chinensis*, and these compounds have demonstrated neuroprotective activity [7]. New triterpenoids and dibenzocyclooctadiene lignans with cytotoxic activities have also been characterized from the leaves of *S. chinensis* [40]. Together with the major lignans, these constituents may contribute to the overall pharmacological profile of *S. chinensis*.

### Pharmacological Activities of Bioactive Components

Although *S. chinensis* contains a variety of bioactive components, the depth of mechanistic evidence is uneven across individual constituents. Among the lignans, schisandrin B (**5**), schisandrin A (**7**), schisandrol A (**6**)/B (**9**), and schisanhenol (**12**) have been repeatedly investigated in pharmacological studies. The following sections emphasize findings supported by experimental data and explicitly identify research gaps where direct compound-specific evidence remains insufficient.

**Antioxidant activity.** Representative lignans from *S. chinensis* exhibit antioxidant activity, but the strength of evidence differs by compound. In liver injury studies, schisandrol B (**9**) reduced iNOS, COX-2, and malondialdehyde (MDA) levels, thereby attenuating lipid peroxidation and inflammatory injury (Table 2) [12]. In an osteoarthritis-related network pharmacology/in vitro study, schisanhenol (**12**) was associated with oxidative stress-related targets, suggesting a possible role in alleviating oxidative injury in chondrocytes (Table 2) [41]. By contrast, direct compound-specific experimental evidence defining antioxidant mechanisms for schisandrin A (**7**) or schisantherin B in the literature covered by this review remains insufficient. Accordingly, these components should currently be regarded as research gaps rather than being assigned mechanisms by analogy to other lignans.

**Anti-tumor effects.** Preclinical studies suggest that some *Schisandra* constituents possess (Table 3) anti-tumor potential [42]. Among the compounds covered in the present review, the clearest direct evidence concerns schisandrin B (**5**), for which cell and animal studies have reported inhibition of proliferation (Table 3), promotion of apoptosis, and suppression of metastasis-related processes [19]. In addition, novel triterpenoids isolated from the stems and leaves of *S. chinensis* have shown inhibitory activity against tumor cell proliferation [7]. However, schisandrol A (**6**) and schisanhenol (**12**) derives mainly from osteoarthritis-related network pharmacology and chondrocyte experiments (Table 3) [41, 43, 44]. These data should not be directly extrapolated as anti-tumor mechanisms. Therefore, direct anti-tumor evidence for schisandrol A (**6**) and schisanhenol (**12**) remains insufficient in the literature covered here.

**Anti-inflammatory effects.** Evidence for anti-inflammatory activity is stronger for schisandrol B (**9**) and schisanhenol (**12**) than for several other individual lignans. In the liver-injury study, schisandrol B (**9**) reduced iNOS and COX-2 expression and was associated with regulation of NF-κB/IL-17-related inflammatory signaling (Table 4) [12]. In the osteoarthritis-related study, schisanhenol (**12**) and related lignans were linked to SRC, EGFR, and MAPK14, with reduced MMP13 production *in vitro* (Table 4) [41]. By contrast, direct compound-specific experimental evidence supporting anti-inflammatory mechanisms for schisandrin A (**7**) remains limited, and the currently available data are insufficient to assign it definite TNF- or VEGF-mediated pathways. Likewise, for schisantherin B, the literature collected in this review does not yet provide sufficiently robust compound-specific mechanistic validation. Thus, these components should presently be described as research gaps rather than as having well-established anti-inflammatory mechanisms.

**Improvement of Sleep/Sedative Effects.** *S. chinensis* has been used since ancient times for its sedative, hypnotic, and mind-calming effects, and it is frequently used clinically to alleviate palpitations and insomnia [1-3]. Among individual lignans, the clearest direct sedative-hypnotic evidence is available for schisandrol A (**6**). Behavioral pharmacology studies showed that schisandrol A (**6**) shortened sleep latency and prolonged pentobarbital-induced sleep duration in mice (Table 5) [16, 17]. For schisantherin B, schisandrin A (**7**), schisandrol B (**9**), and schisanhenol (**12**), however, the currently available literature included in this review does not provide compound-specific experimental evidence sufficient to define definite sleep-improving mechanisms.

Existing supportive data mainly derive from studies of standardized lignan extracts or network pharmacology rather than direct validation of individual compounds. For example, a standardized Schisandra lignan extract showed anxiolytic effects and altered cortical monoamine and corticosterone levels in restraint-stressed mice (Table 5) [18]. Moreover network pharmacology suggested that Schisandra constituents may interact with sleep-wake-cycle-related targets (Table 5) [45]. These findings are valuable at the extract or systems level, but they should not be used to directly infer that schisantherin B, schisandrin A (**7**), schisandrol B (**9**), or schisanhenol (**12**) each possesses a confirmed hypnotic mechanism. Further compound-specific pharmacological studies are still needed.

## Conclusion and Prospects

In summary, *Schisandra chinensis* is an important medicinal and edible resource with a complex chemical composition and broad pharmacological potential. The present review indicates that lignans are the most representative active constituents, while polysaccharides, volatile oils, triterpenoids, and other components also contribute to its biological profile. Current studies support activities including antioxidation, anti-inflammatory effects, anti-tumor effects, hepatoprotection, neuroprotection, sleep improvement, immunomodulation, and regulation of glucose and lipid metabolism. However, when the evidence is examined at the level of individual compounds, the strength of support is uneven: direct experimental evidence is relatively clearer for schisandrin B (**5**) in anti-tumor studies, schisandrol B (**9**) and schisanhenol (**12**) in selected oxidative stress or inflammatory models, and schisandrol A (**6**) in sedative-hypnotic studies, whereas several other reported activities still lack sufficient compound-specific mechanistic validation.

Future research should further clarify the synergistic or antagonistic relationships among multiple components and establish stronger links between chemical composition, quality markers, and pharmacological effects. More systematic pharmacokinetic, toxicological, and *in vivo* efficacy studies are needed to elucidate the absorption, distribution, metabolism, and excretion of key constituents and to support rational dosage and safety evaluation. In addition, high-quality clinical studies, standardized quality-control strategies, and sustainable resource utilization should be strengthened to promote the translation of *S. chinensis* from traditional use to evidence-based application. Importantly, future reviews and mechanistic investigations should clearly distinguish between conclusions supported by direct experimental evidence and genuine research gaps, so that compound-specific activities are not overstated on the basis of extract-level findings, network pharmacology, or structural analogy alone.

## Figures and Tables

**Table 1:** Chemical structures of the main bioactive components.

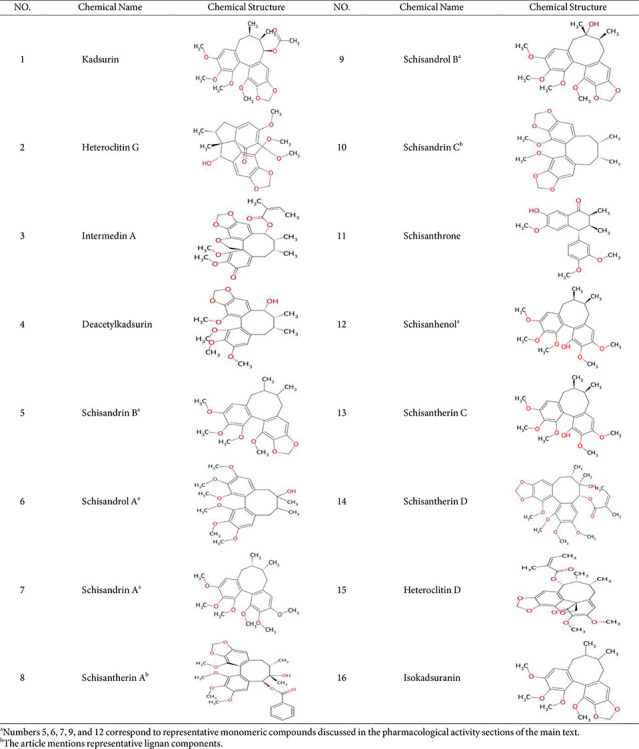

**Table 2:** Antioxidant effects and mechanisms of representative active components from *Schisandra chinensis*.

Component	Pharmacological Effect	Mechanism of Action	Ref.
Schisandrol B (**9**)	Antioxidant activity	Reduced iNOS^a^, COX-2^a^, and MDA^a^ levels in liver injury models to alleviate oxidative/inflammatory injury.	[12]
Schisanhenol (**12**)	Antioxidant activity	Associated with oxidative stress targets in osteoarthritis-related networks, but with limited model validation^b^.	[41]
Schisandrin A (**7**) / Schisantherin B	Antioxidant activity	Research Gap.	[41]

^a^iNOS, malondialdehyde; COX-2, cyclooxygenase-2; MDA, inducible nitric oxide synthase.

^b^There is limited experimental validation obtained from specialized oxidative stress models; direct validation of network pharmacology data is lacking.

**Table 3:** Anti-tumor effects and mechanisms of active components from *Schisandra chinensis*.

Component	Mechanism of Action	Ref.
Schisandrin B (**5**)	Preclinically inhibits proliferation, promotes apoptosis and suppresses metastasis.	[19]
Schisandrol A (**6**) / Schisanhenol (**12**)	Evidence is insufficient. Research Gap.	[41, 43, 44]
Novel triterpenoids (SNTs^a^)	Inhibit tumor cell proliferation in preclinical assays.	[7]

^a^SNTs, schinortriterpenoids.

**Table 4:** Anti-inflammatory effects and mechanisms of active components from *Schisandra chinensis*.

Component	Mechanism of Action	Ref.
Schisandrol B (**9**)	Reduced iNOS and COX-2 expression, regulated NF-κB^a^/IL-17^a^ signaling in liver injury models.	[12]
Schisanhenol (**12**)	Linked to SRC, EGFR, and MAPK14, reduces MMP13^b^ production in osteoarthritis *in vitro* models.	[41]
Schisandrin A (**7**)	Research Gap; TNF^c^/VEGF^c^ pathway assignment needs further validation.	[41]
Schisantherin B	Research Gap.	[41]

^a^NF-κB, nuclear factor-kappa B; IL-17, interleukin-17.

^b^MMP13, matrix metalloproteinase 13.

^c^TNF, tumor necrosis factor; VEGF, vascular endothelial growth factor.

**Table 5:** Neuroprotective and sleep-improving effects and mechanisms of active components from *Schisandra chinensis*.

Component	Mechanism of Action	Ref.
Schisandrol A (**6**)	Shortened sleep latency^a^ and prolonged pentobarbital-induced sleep duration in mice.	[16, 17]
Representative lignans other than schisandrol A (**6**)	Research Gap.	[18, 45]

^a^Sleep latency: time to fall asleep.

## References

[1] Rybnikář M, Šmejkal K, Žemlička M (2019). *Schisandra chinensis* and its phytotherapeutical applications. Michal Rybnikář, Karel Šmejkal, Milan Žemlička. Ceska Slov. Farm..

[2] Szopa A, Ekiert R, Ekiert H (2017). Current knowledge of *Schisandra chinensis* (Turcz.) Baill. (Chinese magnolia vine) as a medicinal plant species: a review on the bioactive components, pharmacological properties, analytical and biotechnological studies. Phytochem. Rev..

[3] Zhang M-Y, Wu H-W, Xu L-P, Yang H-J (2018). Pharmacological effect of *Schisandrae chinensis* Fructus and relative active components on cardiovascular and cerebrovascular diseases. Zhongguo Zhong yao Za Zhi.

[4] Yang K, Qiu J, Huang Z, Yu Z, Wang W, Hu H (2022). A comprehensive review of ethnopharmacology, phytochemistry, pharmacology, and pharmacokinetics of *Schisandra chinensis* (Turcz.) Baill. and *Schisandra sphenanthera* Rehd. et Wil. J. Ethnopharmacol..

[5] Li Z, He X, Liu F, Wang J, Feng J (2018). A review of polysaccharides from *Schisandra chinensis* and *Schisandra sphenanthera* : properties, functions and applications. Carbohydr. Polym..

[6] Ji R, Wang Z, Kuang H (2024). Extraction, purification, structural characterization, and biological activity of polysaccharides from *Schisandra chinensis*: a review. Int. J. Biol. Macromol..

[7] Xu Z-Q, Li S, Long G-Q, Wang D-D, Guo Z-F, Yang Y-C (2023). Wunorlactones A-G, seven undescribed 7/8/5 and 7/8/3 carbon skeleton schinortriterpenoids from the stems and leaves of *Schisandra chinensis*, and their neuroprotective activities. Phytochemistry.

[8] Jia M, Zhou L, Lou Y, Yang X, Zhao H, Ouyang X (2023). An analysis of the nutritional effects of *Schisandra chinensis* components based on mass spectrometry technology. Front. Nutr..

[9] Xu M, Zhang X, Ren F, Yan T, Wu B, Bi K (2019). Essential oil of *Schisandra chinensis* ameliorates cognitive decline in mice by alleviating inflammation. Food Funct..

[10] Zhang S, Liu D, Guo D, Zhao C, Shi Y, Zou J (2025). Pharmacodynamic effects and mechanism of Pueraria lobata-*Schisandra chinensis* combination in the treatment of alcoholic liver disease. Fitoterapia.

[11] Li B, Xiao Q, Zhang J, Wang Y, Liu J, Zhang B (2023). Exploring the active compounds and potential mechanism of the anti-nonalcoholic fatty liver disease activity of the fraction from *Schisandra chinensis* fruit extract fraction based on multi-technology integrated network pharmacology. J. Ethnopharmacol..

[12] Xu G, Lv X, Feng Y, Li H, Chen C, Lin H (2021). Study on the effect of active components of *Schisandra chinensis* on liver injury and its mechanisms in mice based on network pharmacology. Eur. J. Pharmacol..

[13] Hong M, Zhang Y, Li S, Tan HY, Wang N, Mu S et al. 2017. A network pharmacology-based study on the hepatoprotective effect of *Fructus Schisandrae*. *Molecules* **22:** 1617. https://doi.org/10.3390/molecules22101617. 10.3390/molecules22101617 28956809 PMC6151775

[14] Fu J, Zhang H, Liu S, Wu J, Zhang Y, Gao Y (2021). An integrated strategy using LC-MS/MS combined with *in vivo* microdialysis for the simultaneous determination of lignans of *Schisandra chinensis* (Turcz.) Baill. Fructus and endogenous neurotransmitters: application in pharmacokinetic and pharmacodynamic studies. Food Funct..

[15] Zhou C, Chen H, Li W, Yang L, Zeng L, Bian Y (2025). Mechanism of sour jujube kernel-five-flavour berry in the treatment of insomnia based on network pharmacology and molecular docking. Curr. Pharm. Design.

[16] Wu Y, Ding C, Liu C, Dan L, Xu H, Li X (2024). Schisandrol A, the major active constitute in *Schisandra chinensis*: a review of its preparation, biological activities, and pharmacokinetics analysis. Am. J. Chin. Med..

[17] Pharmacology; Findings from Shenyang Pharmaceutical University Provide New Insights into Pharmacology (Pharmacological evaluation of sedative and hypnotic effects of schizandrin through the modification of pentobarbital-induced sleep behaviors in mice). Biotech Week, 2015. 10.1016/j.ejphar.2014.09.012 25446916

[18] Chen W-W, He R-R, Li Y-F, Li S-B, Tsoi B, Kurihara H (2011). Pharmacological studies on the anxiolytic effect of standardized *Schisandra lignans* extract on restraint-stressed mice. Phytomedicine.

[19] Fang Y, Pan J, Wang P, Wang R, Liang S (2025). A comprehensive review of Schisandrin B's preclinical antitumor activity and mechanistic insights from network pharmacology. Front. Pharmacol..

[20] Yoo A, Ahn J, Kim MJ, Seo H-D, Hahm J-H, Jung CH (2022). Fruit of *Schisandra chinensis* and its bioactive component schizandrin B ameliorate obesity-induced skeletal muscle atrophy. Food Res. Int..

[21] Yeon M, Choi H, Jun H-S (2020). Preventive effects of schisandrin A, a bioactive component of *Schisandra chinensis*, on dexamethasone-induced muscle atrophy. Nutrients.

[22] Deng Y-Y, Ma X-Y, He P-F, Luo Z, Tian N, Dong S-N (2025). Integrated UPLC-ESI-MS/MS, network pharmacology, and transcriptomics to reveal the material basis and mechanism of *Schisandra chinensis* Fruit Mixture against diabetic nephropathy. Front. Immunol..

[23] Qiao Z, Du X, Zhuang W, Yang S, Li H, Sun J (2020). *Schisandra chinensis* acidic polysaccharide improves the insulin resistance in type 2 diabetic rats by inhibiting inflammation. J. Med. Food.

[24] Zhang Z, Yang W, Chen J, Chen X, Gu Y (2025). Efficacy and mechanism of Schisandra chinensis active component Gomisin A on diabetic skin wound healing: network pharmacology and *in vivo* experimental validation. J. Ethnopharmacol..

[25] Yan T, Wang N, Liu B, Wu B, Xiao F, He B (2021). *Schisandra chinensis* ameliorates depressive-like behaviors by regulating microbiota-gut-brain axis via its anti-inflammation activity. Phytother. Res..

[26] Yang H-J, Zhang T, Kim M-J, Hur H-J, Wu X, Jang D-J (2024). Efficacy and mechanism of *Schisandra chinensis* fructus water extract in alzheimer's disease: insights from network pharmacology and validation in an amyloid-β infused animal model. Nutrients.

[27] Fan Y, Wang A, Liu Z, Xing J, Zheng Z, Song F (2024). Integrated spatial metabolomics and network pharmacology to explore the pharmacodynamic substances and mechanism of Radix ginseng-*Schisandra chinensis* Herb Couple on Alzheimer's disease. Anal. Bioanal. Chem..

[28] Hao W, Chen J, Zhang Y, Mou T, Wang J, Zhang C (2023). Integration of metabolomics and network pharmacology to validate the mechanism of *Schisandra chinensis* (Turcz.) Baill *Acorus tatarinowii* Schott ameliorating the Alzheimer's disease by regulating the aromatase activity to affect local estrogen in brain of AD model rats. Arab J. Chem..

[29] Wang A, Shi M, Xing J, Liu S, Liu Z, Song F (2022). Treatment effects of Radix ginseng-*Schisandra chinensis* herb pair on Alzheimer's disease: an investigation of MS-based metabolomics investigation. J. Pharm. Biomed. Anal..

[30] Chen J, Hao W, Zhang C, Cui M, Sun Y, Zhang Y (2022). Explore the therapeutic composition and mechanism of *Schisandra chinensis* acorus tatarinowii schott on alzheimer's disease by using an integrated approach on chemical profile, network pharmacology, and UPLC-QTOF/MS-based metabolomics analysis. Oxid. Med. Cell. Longev..

[31] Bansal S, Pratiksha K, Gaur R, Gupta S, Jadaun VP, Kumari V (2024). Comprehensive review on Schisandra chinesis. Pharmacol. Res. Modern Chinese Med..

[32] Zhang M, Xu L, Yang H (2018). *Schisandra chinensis* fructus and its active ingredients as promising resources for the treatment of neurological diseases. Int. J. Mol. Sci..

[33] Wen Y, Fang X, Bai M, Miao M. 2017. Study on the Modern Application of *Schisandra chinensis*. https://doi.org/10.12783/dtbh/mshh2017/13431. 10.12783/dtbh/mshh2017/13431

[34] Jiang P, Lu Y, Chen D (2016). Authentication of *Schisandra chinensis* and *Schisandra sphenanthera* in Chinese patent medicines. J. Pharm. Biomed. Anal..

[35] Piao J, Liu L, Wang S, Shang H-B, He M, Quan N (2018). Magnetic separation coupled with high-performance liquid chromatography-mass spectrometry for rapid separation and determination of lignans in *Schisandra chinensis*. J. Sep. Sci..

[36] Guo YX, Han J, Zhang DY, Wang LH, Zhou LL (2013). Aqueous two-phase system coupled with ultrasound for the extraction of lignans from seeds of *Schisandra chinensis*(turcz.) Baill [J]. Ultrason. Sonochem..

[37] Lv X, Xu Z, Xu G, Li H, Wang C, Chen J (2020). Investigation of the active components and mechanisms of *Schisandra chinensis* in the treatment of asthma based on a network pharmacology approach and experimental validation. Food Funct..

[38] Yuan R, Tao X, Liang S, Pan Y, He L, Sun J (2018). Protective effect of acidic polysaccharide from *Schisandra chinensis* on acute ethanol-induced liver injury through reducing CYP2E1-dependent oxidative stress. Biomed. Pharmacother..

[39] Xu SQ, Meng X, Yang X, Bu G-T, Wang S-T, Li-M-M, et al. 2026. Triterpenoids in the leaves of *Schisandra chinensis* (Turcz.) Baill. treated AD by inducing mitophagy through activation of MCL-1. *Food Biosci.* **79:** 108674-108674. https://doi.org/10.1016/j.fbio.2026.108674. 10.1016/j.fbio.2026.108674

[40] Zhang Y-Q, Liu Y, Wen B, Wang S-Y, Jiang P, Wu J-T (2023). New triterpenoids and a dibenzocyclooctadiene lignan from the leaves of *Schisandra chinensis*. Phytochem.Lett..

[41] Min L, Wu Y, Cao G, Mi D, Chen C (2021). A network pharmacology strategy to investigate the anti-osteoarthritis mechanism of main lignans components of *Schisandrae* Fructus. Int. Immunopharmacol..

[42] Mokhtari T, Kenawy AEME. 204. Molecular mechanisms of *Schisandra chinensis* in treating depression-neuropathic pain comorbidity by network pharmacology and molecular docking analysis. *Neuroscience* **555:** 92-105. https://doi.org/10.1016/j.neuroscience.2024.07.023. 10.1016/j.neuroscience.2024.07.023 39032805

[43] Jeong J-W, Lee HH, Choi E-O, Lee KW, Kim KY, Kim SG (2015). *Schisandrae* fructus inhibits il-1β-induced matrix metalloproteinases and inflammatory mediators production in SW1353 Human chondrocytes by suppressing NF-κB and MAPK activation. Drug Dev. Res..

[44] Han SJ, Jun J, Eyun S-I, Lee C-G, Jeon J, Pan C-H (2021). Schisandrol a suppresses catabolic factor expression by blocking NF-κB signaling in osteoarthritis. Pharmaceuticals.

[45] Liang K, Qiao T, Sun N, Jiang N, Li F, Jiang H (2024). Mechanism of *Schisandra chinensis* in treatment of insomnia by sleep-wake cycle based on network pharmacology. Pharmacogn. Mag..

